# Chronic occupational exposures can influence the rate of PTSD and depressive disorders in first responders and military personnel

**DOI:** 10.1186/s13728-016-0049-x

**Published:** 2016-07-15

**Authors:** Anthony Walker, Andrew McKune, Sally Ferguson, David B. Pyne, Ben Rattray

**Affiliations:** University of Canberra Research Institute for Sport and Exercise, University of Canberra, Canberra, Australia; Australian Capital Territory Fire & Rescue, Canberra, Australia; Discipline of Sport and Exercise Science, Faculty of Health, University of Canberra, Canberra, Australia; Appleton Institute, School of Human Health and Social Sciences, Central Queensland University, Adelaide, Australia; Department of Physiology, Australian Institute of Sport, Canberra, Australia

**Keywords:** Cytokines, IL-6, Mood disorders, Firefighters, Military, Inflammation, Sickness behaviour

## Abstract

**Background:**

First responders and military personnel experience rates of post-traumatic stress disorder (PTSD) far in excess of the general population. Although exposure to acute traumatic events plays a role in the genesis of these disorders, in this review, we present an argument that the occupational and environmental conditions where these workers operate are also likely contributors.

**Presentation of the hypothesis:**

First responders and military personnel face occupational exposures that have been associated with altered immune and inflammatory activity. In turn, these physiological responses are linked to altered moods and feelings of well-being which may provide priming conditions that compromise individual resilience, and increase the risk of PTSD and depression when subsequently exposed to acute traumatic events. These exposures include heat, smoke, and sleep restriction, and physical injury often alongside heavy physical exertion. Provided the stimulus is sufficient, these exposures have been linked to inflammatory activity and modification of the hypothalamic–pituitary axis (HPA), offering a mechanism for the high rates of PTSD and depressive disorders in these occupations.

**Testing the hypothesis:**

To test this hypothesis in the future, a case–control approach is suggested that compares individuals with PTSD or depressive disorders with healthy colleagues in a retrospective framework. This approach should characterise the relationships between altered immune and inflammatory activity and health outcomes. Wearable technology, surveys, and formal experimentation in the field will add useful data to these investigations.

**Implications of the hypothesis:**

Inflammatory changes, linked with occupational exposures in first responders and military personnel, would highlight the need for a risk management approach to work places. Risk management strategies could focus on reducing exposure, ensuring recovery, and increasing resilience to these risk contributors to minimise the rates of PTSD and depressive disorders in vulnerable occupations.

## Background

First responders and military personnel work in physically demanding settings characterised by traumatic events and personal danger, often coupled with environmental and occupational stressors. Possibly, as a consequence of ongoing exposure to traumatic events, first responders and military personnel experience relatively high levels of depression and post-traumatic stress disorder (PTSD) [1, 2]. These outcomes can be initiated by exposure to a range of internal and external cues, and are often characterised by avoidance and periods of hyper arousal [3]. Currently, most treatment programs for PTSD focus on the response to sudden traumatic events. However, it is unclear whether acute exposure to traumatic events is the sole reason for the high prevalence of depression and PTSD in first responders and military personnel. While research efforts have generally focussed on increasing psychological resilience, the underlying physiological mechanisms that predispose individuals to developing depressive disorders or PTSD remain unclear. In this review, we examine possible links between occupational exposures to heat and smoke, sleep restriction, and chronic physical stress that could predispose emergency and military personnel to depression or PTSD.

Exposure to maimed or fatally injured persons is recognised as a substantial contributor to increased prevalence of health care utilisation and mental health care access by first responders [4] and military personnel. Specifically, exposure to acute traumatic events is associated with ~13–18 % of first responders experiencing PTSD following events, such as large-scale emergencies [1], terrorist attacks [5, 6], natural disasters [7, 8], and industrial accidents [2, 9]. Unlike the general population, many first responders and military personnel will be exposed to traumatic events numerous times during their career. Thus, possibly, as a result of multiple exposures, lifetime prevalence of PTSD in first responders can be as high as 32 % [2], compared with estimates in the general population of 6–14 % [1].

Not all first responders or military personnel experience PTSD after exposure to stressful events [10, 11]. For example, despite similar exposure to war casualties, US army medics returning from Iraq displayed lower rates of PTSD or depression than soldiers [1]. Medics reported frequent exposure to combat (28 %), to injured or dead friendly forces (62 %), perceptions of personal danger (38 %), and potential death (23 %), all which are psychological stressors. Although this incidence (20–60 %) compares with up to 90 % of soldiers reporting frequent combat exposure [12], the higher rates of depressive disorders in soldiers appear disproportionally high given that medics are ~17 times less likely to develop symptoms of PTSD than soldiers in the same theatre of battle [12]. It may also be that those individuals self-selecting to fulfil the role of medics may be more psychologically resilient than soldiers. Regardless, a clear differentiation between soldiers and medics in the operational requirements of their roles, in particular the exposure to environmental conditions and physical exertion and trauma, likely occurs. Thus, it appears that exposure to traumatic events may not be solely responsible for the rates of PTSD and depression in military or first responder populations, and other environmental or physiological factors might mediate the increased risk of these disorders.

## Presentation of the hypothesis

**Fig. 1:**
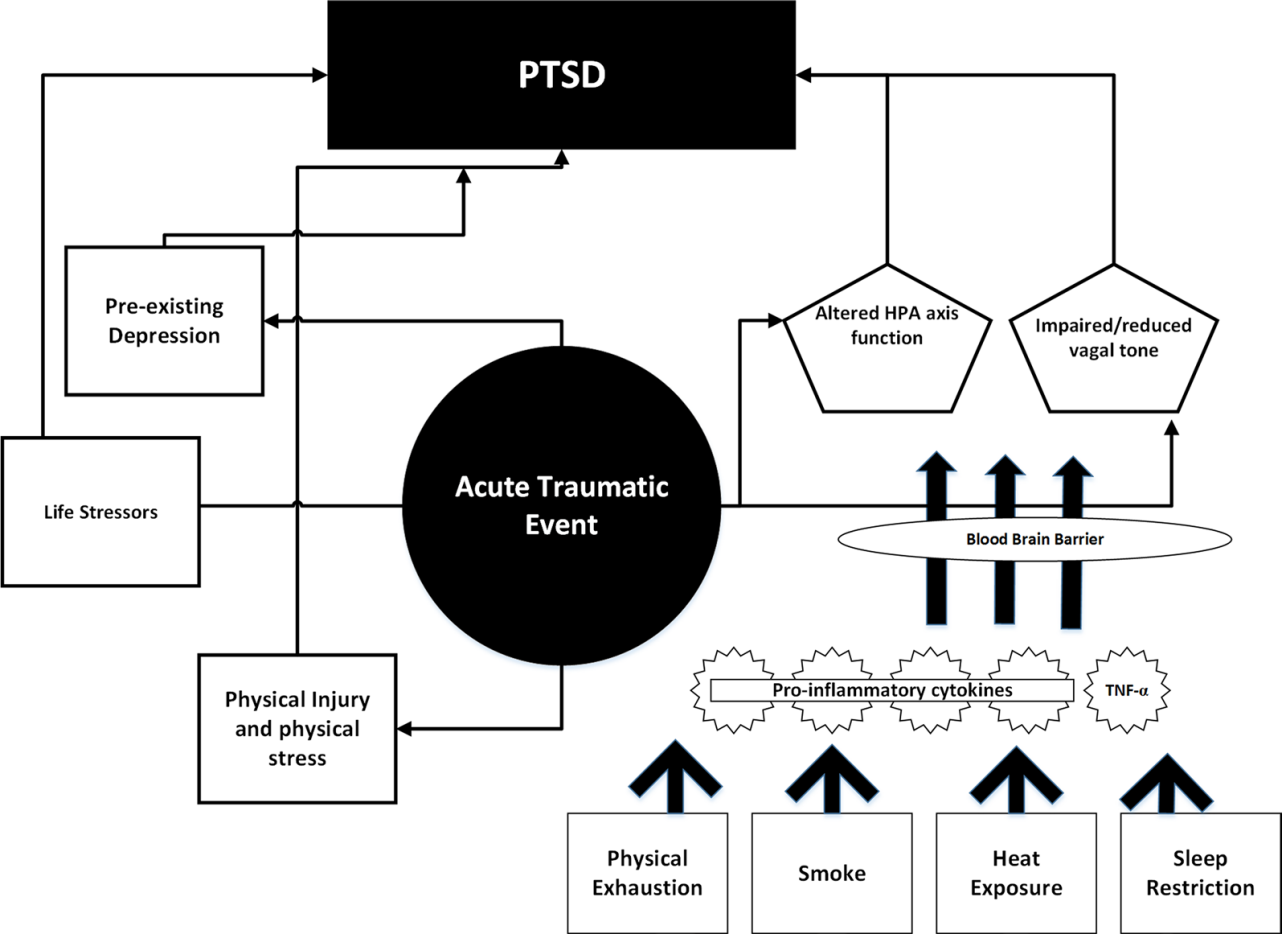
Working model where pre-priming environmental and occupational work factors may predispose individuals to PTSD following exposure to acute traumatic events. By providing a pre-priming effect, it is likely that individual resilience may be compromised when faced with traumatic events at work

Although depression and PTSD are complex multi-faceted conditions or perhaps, set of conditions, inflammation plays a role in many depressive states. Both acute bouts of inflammation, as well as chronic low-grade inflammation, have been linked to depressive states, and in vulnerable individuals, the reduction of inflammation is likely to improve psychological and general well-being. First responders and military personnel are regularly exposed to environmental and occupational stressors that present as pro-inflammatory stimuli. Thus, we propose that these exposures provide a priming stimulus (Fig. 1), which predisposes individual workers to a higher risk of depressive disorders following acute traumatic events.

## Pathophysiology of depression

### Sickness behaviours

Sickness behaviours are common responses observed during periods of illness and injury. Sickness behaviour was originally described in ill animals with reduced activity and sleep, feeding, and grooming and social interactions [13] akin to signs of depression [14]. This grouping of behaviours is believed to have been conserved throughout evolution [13, 15] most likely as a generalised adaptation to infection and injury. It makes sense that the body has a built-in signal to alter behaviour and conserve energy when under stress, preserving available resources and energy to heal. These behaviours are not merely reflexive reactions to “illness” either, but represent a central motivational state [16], that assists the organism in recovery, and potentially protects others in the group [17].

### Inflammation

It was long believed that the blood–brain barrier (BBB) provided an impenetrable barrier to protect the brain and wider central nervous system (CNS) from systemic molecules that are potentially damaging or responsible for behaviour change. However, more recent evidence indicates that acute or chronically elevated levels of circulating cytokines can cross the BBB through a variety of mechanisms [18] and signal the brain via both direct and indirect pathways. This communication can lead to neural and associated behaviour changes, such as feelings of tiredness and reduced motivation [19]. Dantzer and colleagues [19] proposed that systemic low-grade inflammation, typically defined by a two- to three-fold increase in plasma concentrations of pro-inflammatory cytokines, such as interleukin (IL)-1, tumour necrosis factor (TNF)-α, and IL-6 [17, 20], may lead to clinical depression in vulnerable individuals [14]. Thus, we argue that systemic chronic low-grade inflammation, possibly occurring as a result of numerous occupational stressors, may prime vulnerable individuals for an elevated risk of depressive disorders, especially when exposed to acute traumatic events.

### Cytokines

Elevated pro-inflammatory cytokines, which, in first responders, may result from environmental exposures [21], have been linked to depression in the acute phase of PTSD [22–24] and during remission [25]. Coupled with increases in acute phase proteins, such as complement protein, C-reactive protein (CRP), and haptoglobin, elevated pro-inflammatory cytokines have been observed in major depression [17, 26]. Specifically, depressed patients can exhibit substantially higher levels of IL-1β and IL-6 than non-depressed controls [27]. Higher levels of IL-6 (24.0 *vs* 14.6 pg ml^−1^) were observed in the cerebrospinal fluid of combat veterans suffering PTSD compared with non-depressed controls [28]. Such observations occur, since some cytokines can cross the BBB rapidly and access the CNS via several routes [18]. For example, TNF-α crosses the BBB within 30 min of exogenous injection [18]. Cytokines can enter the CNS via pathways that include direct access to brain structures, either using a saturable influx transport system (for IL-1α and β, IL-6 and TNF-α), or simple diffusion at the level of circumventricular organs, where the BBB is incomplete [29, 30]. Cytokines may also reach the CNS indirectly via activation of afferent neurons [31] of the vagus nerve where they promote transcription and translation of other cytokines within the CNS [32]. A systemic increase in pro-inflammatory cytokines and their movement to the brain thus conveys information about metabolic, gastrointestinal, and cardiovascular challenges to brain regions that mediate stress-related behaviour [17], in particular, sickness depressive- and anxiety-like behaviours [33].

Specific receptors for IL-1α, IL-6, and TNF-α have a discrete distribution in the brain. For example, IL-1α and IL-6 receptors are abundant in the hypothalamus [31] and hippocampal areas [34]. Blocking IL-1α receptors in the brain can prevent some of the sickness responses to peripheral administration of cytokines [35]. Further evidence of the role of these cytokines comes in the form of a recent meta-analysis showing depressed patients exhibit two-fold higher levels of IL-1β and IL-6 than non-depressed controls [36]. IL-1β and IL-6 can activate several discrete hypothalamic nuclei, which may account for many of the sickness-related behavioural changes, including hunger, thirst, sleep, reduced libido, and changes to resting body core temperatures [31]. Similarly, administration of certain cytokines, including IL-1β and IL-6, directly into the brain produces many or all of the sickness responses [35, 37]. Elevated cytokines may also interfere with cognitive processes, such as loss of attention and certain types of memory, an inability to concentrate at work, and reduced learning retention [31]. Any alterations of attention, memory, and/or learning may then, subsequently, decrease the ability of affected individuals to process their experience and reduces their long-term resilience [38].

One of the cytokine/CNS communication pathways for inflammation includes a dysregulated “fight-or-flight” response involving the autonomic nervous system and the hypothalamic–pituitary–adrenal (HPA) axis [39]. Activation of the HPA axis and sympathetic nuclei results in binding of cytokines in the hypothalamus, synthesis, and release of glucocorticoids [40] and increased levels of circulating catecholamines and cortisol [34, 41]. Compared with the general population, studies evaluating epinephrine and norepinephrine in PTSD have found increased resting levels [27, 42], while others have reported an exaggerated response after trauma-related stimulation [43]. However, the association of cortisol levels with traumatic exposures and PTSD is still not well understood. The hippocampus plays a role in modulating activity of the HPA axis [40] via a negative feedback system that involves glucocorticoids binding to receptors [44] in the hippocampus [45, 46]. In the cases where HPA axis activation is sustained, possibly due to repeated stress, ongoing elevated glucocorticoid levels have been associated with mood impairments, including depression and PTSD [40, 47]. Clinical studies in traumatized individuals with PTSD have demonstrated a reduction in volume of the hippocampus with associated memory and learning deficits, indicating the neurotoxicity of cortisol [41]. However, while it may be assumed that individuals who develop PTSD following exposure to trauma might have sustained levels of cortisol [48], research has reported that a lower cortisol response to trauma is a predictor of subsequent PTSD symptoms in police officers and firefighters [49]. Impaired hippocampus function, related to repeat occupational stressors and chronically elevated cortisol levels, may be responsible for this inadequate cortisol response to trauma [48]. Consequently, in individuals with PTSD, or those susceptible to PTSD, an inadequate cortisol response following trauma may delay recovery by disrupting biological homeostasis acutely and possibly interfere with the interpretation of stressful information resulting in chronic disruptions in memory integration.

In addition to the measurement of catecholamines in PTSD, activity of the autonomic nervous system can be determined using heart rate variability (HRV) assessments [50, 51]. High HRV is associated with increased vagal/parasympathetic nervous system activity, whilst low HRV is linked with a withdrawal of vagal activity and a shift towards sympathetic nervous system dominance [51]. In addition to understanding the parasympathetic/sympathetic balance, HRV indices also provide information about an individual’s ability to regulate inflammation. The vagus nerve plays an important role in regulating inflammation [52]. Specifically, it has been shown that cell production of inflammatory cytokines can be blocked via vagal nerve activity and that low HRV, indicative of vagal withdrawal, may reflect a reduced ability to regulate immune responses [52]. Recently, lower pre-deployment HRV (indicative of vagal withdrawal) reported in active-duty US marines [53] was associated with an increased prevalence of post-deployment PTSD [54]. Further research is required to characterise the acute and chronic relationships between HRV, inflammation, depression, and/or PTSD. Screening of first responders at varying stages in their career or during military pre-deployment activities might provide stronger evidence of this link.

## Occupational exposures

Many military personnel and first responders work in hostile environments often characterised by high levels of heat [55, 56] and smoke [57], with altered sleep patterns [58] and risk of sustaining a physical injury [59]. Furthermore, work tasks, including wildfire suppression, complex technical rescues, and patrolling on foot (soldiers), can involve prolonged, strenuous bouts of physical activity. When this work is repeated over multiple days, as often occurs during responses to natural disasters and extended military deployments, individuals are also likely to experience reduced recovery and rest [58]. Collectively, these challenges could result in fatigue and exhaustion, contributing to a state of chronic low-grade systemic inflammation and/or altered immune status. We propose a model where chronic low-grade systemic inflammatory changes arising from prolonged occupational exposures in vulnerable first responders and military personnel may predispose this cohort to PTSD when exposed to subsequent traumatic events.

### Heat

A growing body of evidence points to links between elevated core temperature (*T*_c_) and increased immune system activity. Elevated levels of circulating leukocytes and platelets, and increased inflammatory activity, have been observed in firefighters following simulated work tasks in the heat [21, 60]. Strenuous physical activity undertaken in hot environments can also increase circulating levels of lipopolysaccharide (LPS) [61]. At rest and when the body is in a thermo-neutral state, the gastrointestinal mucosa protects the systemic environment from ingress of LPS, an endotoxin [62–65]. At low levels, the liver protects the body by eliminating LPS from circulation [66]. However, when T_c_ increases substantially following work in the heat, intestinal permeability can increase, leading to accelerated migration of LPS into the circulatory system [61, 67, 68]. When LPS concentration rises rapidly in the blood, a range of pro-inflammatory responses, including IL-1, TNF-α, and IL-6, are triggered [69–71]. Rising levels of LPS in the blood may then invoke a pathological systemic inflammatory response particularly in the presence of pre-existing suppressed immune surveillance for LPS, as has been shown in military personnel [72, 73].

Firefighters, tactical police, explosive ordinance disposal (EOD) operators, and soldiers wear personal protective clothing (PPC) and accoutrements. This protective clothing restricts movement [74] and can add substantial mass [75] to the ambulating individual. Furthermore, the need to provide first responders with protection from steam, chemicals, and biological attacks requires significant levels of encapsulation [76] along with protective equipment worn by soldiers and, increasingly, specialist, and general duties police officers. Wearing PPC (particularly “bunker gear”, hazardous material (HAZMAT) protection, “bomb suits”, and body armour) can create an uncompensable environment for operators resulting in an elevated *T*_c_. In work settings, PPC, thus, contributes to increases in *T*_c_ two ways: (i) via increasing metabolic heat production related to weight and binding [74], and (ii) limited body heat dissipation through reduced evaporative capacity [77].

While recommendations regarding post-incident rehabilitation and cooling are evolving [78, 79], it is unclear how many first response agencies or military organisations employ evidence-based practices following work in the heat [80]. As a result, workers may be redeployed following work tasks with elevated *T*_c_, possibly above recommended limits. Sustained elevations in *T*_c_ during these long duration exposures likely lead to increased immune and inflammatory activity, with post-heat inflammation observed for up to 24 h following simulated firefighting tasks in hot environments [21, 81, 82]. Given the recognised links between inflammation and depression, regular ongoing exposure to heat in the workplace, or sustained *T*_c_ elevations, may further contribute to the high rate of depression and PTSD in these populations if untreated.

### Smoke and toxic gases

In addition to working in the heat, firefighters, in particular, face smoke, and toxic gases during fire suppression activities. Exposure to fine particulates, such as those in fire smoke and diesel exhausts from plant and machinery, promotes inflammation in the lungs [57, 83]. Smoke-related inflammation in the lungs has subsequently been associated with acute increases in circulating IL-6 and TNF-α [57]. Respiratory symptoms at work were more prevalent in male Swiss firefighters compared with a control group from the general population. In this study, atopic diseases (including asthma and allergic rhinitis) were present in 51 % of firefighters compared with 32 % in a control group [84]. Recurrent depression and atopic diseases appear to be associated with atopy-related pro-inflammatory cytokines that play a role in the underlying mechanism [84].

While urban firefighters are generally protected from smoke and particulates by wearing self-contained breathing apparatus (SCBA), wildland firefighters generally operate without such protective equipment for extended durations, resulting in increased exposure to particulate matter, respiratory irritants, and systemic toxins, including asphyxiants [83, 85]. Furthermore, despite the protection offered to firefighters by wearing an SCBA, its effectiveness may be reduced during overhaul activities, when firefighters may doff their equipment and are subsequently exposed to off-gassing from burnt materials [86]. Improper supervision or poorly designed work practices may also see firefighters not fully protected by their SCBA, which must be considered by incident controllers when conducting fire suppression activities. The risk of contamination may be further increased when firefighters fail to engage in proper decontamination procedures at fire scenes, including entering rehabilitation sectors or travelling back to their station in contaminated PPC and SCBA.

### Sleep restriction

Emergency first responder agencies provide a 24/7 response, requiring personnel to work and/or be on-call around the clock. Shiftwork and on-call work both cause well-described disruptions to sleep and the circadian rhythm [87–90]. The circadian rhythm in the brain drives sleep during the night hours and activity during the day hours. Shiftwork, and in particular night work, affects the circadian rhythm in two ways: (i) fighting to remain awake and alert at night is associated with reduced alertness and impaired performance, while (ii) fighting to fall and stay asleep during the day is associated with shorter and less restorative sleep. Thus, the shift worker deals with both compromised sleep and circadian misalignment [70–73]. The endocrine system also responds to sleep disturbance in the form of elevated cortisol [91] and downstream effects reducing HRV [92].

The effect of sleep deprivation on systemic inflammation and inflammatory markers is unclear. Some studies have demonstrated little effect of sleep deprivation on circulating leukocyte counts [93, 94] with effects similar to that of intense exercise (14), making an individual more susceptible to viral infections, such as the common cold and influenza (15,16). In contrast, one night of sleep deprivation increased circulating leukocyte counts in another study [95]. Furthermore, higher pro- and anti-inflammatory responses have been reported in healthy participants in laboratory studies involving sleep restriction or total sleep deprivation [78, 84, 85]. Using a more ecologically valid, field-simulated protocol, Wolkow and colleagues examined the sleep restriction which occurs on multi-day wildfire deployments and during 24/7 emergency responses [96]. A two-fold increase in resting levels of circulating pro-inflammatory IL-6 and a 14 % increase in cortisol were observed in wildland firefighters following successive days of physical work [58, 96]. These outcomes were mirrored in young male footballers who, after completing high-intensity work tasks following partial sleep deprivation, also demonstrated significant increases in IL-6 (~25 %), TNF-α (~69 %), and growth hormone (~25 %) compared with undertaking training in a rested state [97, 98].

Sleep disruption is associated with depressive disorders [99, 100], but the causal relationship is not well defined. It is reasonable to assume that challenges associated with mental health issues may precipitate sleep disruption. A longitudinal study in military personnel points to sleep disturbance as a precursor to PTSD symptoms and depression [101] and inflammation associated with sleep disruption may play a role. Healthy men exposed to one night of sleep deprivation secreted higher daytime levels and lower night time levels of IL-6 than following a normal night’s sleep. Furthermore, data from the control day indicated that better sleep quality was associated with lower daytime levels of IL-6 [102]. Inflammatory responses are linked to sleep disruption as well as depression, fatigue, sensitivity to pain, and cognitive impairments [103], and may precipitate adverse mood and mental health outcomes [96, 97, 104, 105].

### Physical exhaustion/overtraining

When first responders and military personnel undertake strenuous physical work over multiple days, recovery may be limited, resulting in a condition similar to the symptoms of overtraining [106]. In athletic populations, overtraining manifests in reduced physical and cognitive performance [107] along with mood changes, excessive fatigue, and frequent illness [108]. High frequency of infection or illness symptoms in overtraining affected athletes is related to altered immunity, including increased inflammation and suppressed mucosal or cell-mediated immunity [109]. The altered immunity in overtraining athletes, or in athletes/workers following strenuous prolonged bouts of exercise, may be related to tissue damage up-regulating pro-inflammatory cytokines. These cytokines increase the risk of infection and/or non-infectious upper-respiratory symptoms [109, 110]. Elevated immune suppression and inflammatory reactions are usually self-limiting, particularly in healthy and well-rested athletic populations. However, when proper recovery is not achieved, as may occur during multi-day deployments, chronic inflammation and immune suppression may occur [109, 111]. The overtraining-driven pro-inflammatory cytokine activation initiates changes in the hypothalamus, mood state, as well as the temperature set point [109], likely exacerbating the negative effects of working in the heat. Thus, efforts to reduce the likelihood of overtraining, or an imbalance between physical exhaustion, fatigue, and recovery, in operational populations may increase the rate of PTSD.

### Physical injury

Soldiers, in particular, face an increased likelihood of physical injury, while on deployment, and are increasingly exposed to the effects of high explosives, which are responsible for ~60 % of modern US combat-related casualties [112]. Ninety percent of combat veterans with more than five episodes report ailments, including neurological deficits and cognitive impairment [112]. Since the earliest days of high-explosive use in combat, soldiers have presented with PTSD symptoms, many previously referred to as “shell shock”. However, until recently, the link between blast-related mild traumatic brain injury (TBI), characterised by altered or lost consciousness for up to 30 min, and PTSD has been largely overlooked. This is despite 44 % of US soldiers in Iraq presenting with PTSD reporting lost consciousness during deployment [113]. Furthermore, personnel exposed to blast-related mild TBI, regularly report persistent symptoms, including impaired concentration, memory problems, depression, and anxiety indicative of structural damage in the brain, including the hippocampus, amygdala, and cortex regions [112, 113]. It is possible, therefore, that an individual’s ability to regulate fear during stressful or traumatic events can be impaired, resulting in insufficient cognitive resources and a greater incidence of PTSD [114]. While not exactly the same, these symptoms are similar to chronic traumatic encephalopathy (CTE), a condition seen in contact sports [112].

In addition to acute and repeated blast-related TBI, a study of Israeli soldiers reported that those who were injured in combat suffered an eight-fold increase in the incidence of PTSD compared with their healthy counterparts [59]. This outcome is consistent with studies of returned soldiers from the Vietnam conflict who presented a two- to three-fold increase in PTSD compared with uninjured personnel [115, 116], particularly should they be exposed to a penetrating brain injury and subsequent traumatic event [113].

In the context of PTSD, changes in the HPA axis arising from physical injury, such as TBI or an impact wound, are likely to play a role in increased incidence of PTSD and depression in returning servicemen and women [17]. Specifically, endocrine disturbances are common after TBI, including HPA axis failure and adrenal insufficiency [117]. Inadequate control of inflammation by the HPA axis, evidenced by a more positive association between cortisol and CRP during stress, can give rise to health problems [118]. Recently, Laurent et al. [119] argued that negative health consequences of chronically high activation or inadequate post-stress recovery in the HPA axis and inflammatory systems are more pronounced when they occur together. Intuitively, therefore, physical injury-induced alterations in the HPA axis and dysregulation of inflammation may present as contributors to depression and PTSD. However, despite post-mortem examinations of TBI-affected soldiers, to date, clinical biomarkers are yet to be established to enable “real-time” monitoring of vulnerable personnel [112].

## Testing the hypothesis

To test the hypothesis presented in the present paper, a case–control approach is suggested that compares individuals with PTSD or depressive disorders with healthy colleagues in a retrospective framework. This framework should aim to characterise the relationships between altered immune and inflammatory activity, HPA activity, and health outcomes. The experimental approach needs to consider the involvement of heat, smoke, sleep restriction, physical exertion, inflammatory activity, and HPA axis modification. Initially, the involvement of these factors could be identified in part by questionnaire, wearable monitoring devices, and formal experimentation. The following list of measures should be useful in evaluating the hypothesis.

### Heat

- Baseline immune, inflammatory, HPA, and HRV markers to assess whether some individuals are more at risk of depression.
- Body composition and fitness assessment.
- The impact of multiple exposures over multiple days.

Limited research exists with first responders and military personnel when they work in the heat; rather data are generally collected in controlled laboratory experiments or simulations in the field. To test the hypothesis that working in the heat elicits immune and inflammatory changes, combining data obtained in the field, during actual operations, with workers compensation data (specifically PTSD and depressive disorder claims) are needed to provide evidence of this possible link. Given the proven validity of relatively easy to deploy monitoring tools, such as ingestible core temperature sensors, heart rate monitors, and other wearables, these can be used during long duration, pre-planned responses. Furthermore, examination of cortisol responses over multiple days may provide further understanding of the role of heat as a stressor contributing to depressive disorders [120].

While recommendations regarding post-incident rehabilitation and cooling continue to evolve [66, 67], it is unclear how many first response agencies or military organisations engage in evidence-based practices for heat-affected workers [68]. For instance, the National Fire Protection Association (NFPA) recommends that firefighters attend recovery areas at emergencies following two work sessions in hot environments [78], an established protocol in other jurisdictions [55]. However, many fire services rely on more traditional passive cooling protocols, namely fluid replacement and shade, as a surrogate for proper active methods resulting in poor cooling rates [80]. Further research into the relationships between post-incident cooling and possible reductions in immune activity should be explored to evaluate whether current cooling practices can be improved.

### Smoke

- Measurement or reporting of off-gassing PPE.
- Comparison of immune and inflammatory responses in wildland compared with urban firefighters wearing SCBA.
- Baseline and ongoing monitoring of firefighters during emergency responses and deployments.

Atmospheric monitoring of operating environments can be easily achieved using the modern technology, including photo-ionisation-detection (PID) devices, and other detectors already used in many urban fire services. This approach can give some clarity to the degree of particulate exposure in the field. Furthermore, laboratory examination of soiled PPE can give further evidence of levels of exposure and also exposure types during fire suppression activities. This data can then be compared with changes to baseline immune and inflammatory responses of firefighters following exposure in the field, particularly in wildland firefighters who generally operate without the protection offered by an SCBA. Furthermore, many urban fire services now test for exposure to carbon monoxide following fire suppression—this will add further information regarding possible exposure limits of firefighters.

### Sleep restriction

- Self-reported assessments of sleep quality and quantity.
- Remote monitoring of sleep using wearable devices.
- Detailed reporting of work, exercise and other activities, and rest times.

We propose that sleep plays a role in both susceptibility to and development of PTSD, as well as being a symptom of PTSD and depressive disorders. Thus, it will be important to collect information on subjective sleep quality and quantity under various operational and rest scenarios. In observational studies, objective sleep information can be collected using wearables and other validated devices to determine the amount of sleep obtained in given opportunities and to identify relationships between sleep and other stressors on negative health outcomes. Organisations and individuals should also be aware of the impact of sleep disorders on sleep quality and quantity, and the acute and long-term implications for performance, health, and well-being [121]. Screening for sleep disorders should be considered part of a fatigue risk management system.

### Physical exertion/overtraining/injury

- Self-reported activity, fatigue, and injury records (e.g., online or smartphone capture).
- Biomarker relationships with resilience (e.g., wearable devices to quantify movements, HRV monitoring, other physiological indices).
- Pre-deployment state and relationship to outcomes (e.g., fitness status and health outcomes post-deployment).

In first responder and military populations, examining the links between acute stressors and exercise, or, perhaps, fitness, as a mediating factor is worthy of investigation. In elite athletic cohorts, HRV monitoring is used to assess training status and the presence of non-functional overreaching [122]. Examining HRV to diagnose fatigue, possibly using short 5-min recordings on awakening in the morning, using a domain index RMSSD (square root of the mean of the sum of the squares of differences between adjacent normal R–R intervals) is considered reliable and practical in athletic populations [123]. This approach could be used during deployments of first responders and soldiers to monitor changes and possibly allow for early interventions. Lower HRV (reduced vagal drive) may function as a predictor of vulnerability to inflammatory-related depression [52] to provide a warning sign in first responders. Previously, changes in HPA function have been observed in combat veterans in the form of low cortisol levels [28, 46] and a disrupted autonomic nervous system (ANS) [47]. Lower HRV has also been associated with PTSD in active-duty US marines [54]. Screening of first responders and military personnel together with chronic monitoring of HRV will provide stronger evidence of this link. Furthermore, examining the combination of appropriate recruitment standards, wellness (fitness) programs, and pre-deployment screenings is likely to play an important role in mitigating the high rates of PTSD and depressive symptoms.

## Implications of the hypothesis

While inflammation is not likely to account for all episodes of depression or PTSD in first responders, an underlying inflammatory state is likely in many circumstances. Identifying workplace risks, particularly those related to physical and environmental stress, in addition to acute traumatic stressors, would be a prudent approach to mitigate the high rates of depression and PTSD in first responders. This approach may involve strategies, such as reducing exposure to environmental and occupational factors, screening or monitoring programs, and education of personnel, supervisors, and management. Building individual resilience is also likely to be a meaningful proactive strategy. Practical interventions in need of further research which may reduce the risk of depressive disorders are summarised in Table 1.

**Table 1:** Practical interventions that may mitigate the effects of acute traumatic events prior to developing PTSD

Strategy	Intervention	Possible outcome?
Increasing resilience	Increase fitness	Reduced relative work intensity for individuals
	Managing fatigue—appropriate work/rest cycles	Reduced fatigue and risk of overtraining
	Psychological screening	At risk, individuals may be removed prior to exposure or proactive, targeted interventions are put in place
	Injury management/rehabilitation	Proper injury management, particularly in the form of early intervention will reduce the duration of injury and possibly related immune changes
Working in the heat/smoke	Appropriate PPC selection for the task	Reduced thermal strain of workers
	Pre-cooling of workers	Reduced thermal strain during work events possibly due to lower peak *T*_c_
	Post-incident cooling	Reduce inflammation to resting state as soon as possible following work. Reduce *T*_c_ rapidly to baseline levels
	Appropriate work/rest cycles	Minimising *T*_c_ rise and subsequent inflammatory activity
Sleep deprivation	Cognitive behaviour therapy	Individuals demonstrating sleeping disorders may improve the quality and duration of sleep
	Sleep hygiene	Individuals will have better strategies for managing sleep in the field and during deployments
	Screening for sleep disorders	Individuals can be targeted for interventions
	Rostering for rest periods	Reduced fatigue of possibly at-risk individuals

### Improving fitness

Properly conditioning workers should enable them to safely complete work tasks in a range of occupational settings [124, 125]. This outcome is likely related to a higher capacity to sustain work, and, therefore, reduced stress given any particular workload. Further to fitness, body composition plays a role in the higher risk of injuries in overweight and obese firefighters [126]. This is an important issue in modern fire services with the rates of overweight and obese firefighters continuing to increase [127, 128]. Although the military services regularly test for the fitness of their personnel, this is, generally, not the case in emergency response agencies [129].

Fitness and improved body composition are associated with a better capacity to tolerate heat [130]. A study of US Marine Corp recruits showed those in poorer physical condition (reflected in slower run times and lower predicted VO_2 max_) were up to six times more likely to suffer an exertional heat illness than their fitter colleagues [131]. Thus, improving the fitness and body composition of workers should improve their resilience to work-related physical stress.

### Post incident rehabilitation—include cooling strategies

To allow for proper post-incident recovery, and reduce the likelihood of operators developing non-functional overreaching, incident controllers must consider rehabilitation when planning for long-duration responses. Reduced sleep is likely to be a contributing factor during multi-day responses. Protecting sleep during incidents can be provided in two ways—adequate time for sleep away from the incident ground between work periods and a suitable sleeping environment which reduces the impact of heat, noise, and light [132]. Hence, addressing sleep quality will likely improve mental health in the field. In relation to post-incident recovery, extended or ad lib night sleep opportunities can promote recovery in a range of outcomes, including performance [133, 134], subjective fatigue, and sleepiness [135]. This process involves nighttime sleep opportunities unrestricted by work requirements following an incident. Rostering guidelines should include a number of days ‘off’ before returning to the incident, or responding to a subsequent incident.

## Conclusion

Elevated rates of depression and PTSD in vulnerable occupational populations, in addition to acute traumatic events, could be linked with exposures in their working environments. Exposure to heat, smoke, injury, repeated physical work, and sleep deprivation appears to elicit inflammatory changes that may be priming first responders and military personnel to react adversely to acute traumatic events increasing the risk of PTSD. These environmental exposures should be considered as part of a risk management approach to mitigating PTSD in these populations. A case–control experimental approach is needed to compare individuals with PTSD or depressive disorders with healthy colleagues for characterising relationships between altered immune and inflammatory activity and health outcomes. Appropriate conditioning and identifying at-risk personnel prior to deployment to physical environments characterised by heat, smoke, physical trauma, and sleep deprivation are priorities for first responder and military organisations.

## Abbreviations

ANS: autonomic nervous system
BBB: blood brain barrier
CNS: central nervous system
CRP: C-reactive protein
CTE: chronic traumatic ecephalopathy
GI: gastrointestinal
EOD: explosive ordinance device
HAZMAT: hazardous materials
HPA: hypothalamic–pituitary–adrenal
HRV: heart rate variability
IL: interleukin
LPS: lipopolysaccharide
NFPA: National Fire Protection Association (USA)
PID: photo-ionisation detector
PPC: personal protective clothing
PTSD: post-traumatic stress disorder
RMSSD: the square root of the mean of the sum of the squares of differences between adjacent normal R–R intervals
SCBA: self-contained breathing apparatus
TBI: traumatic brain injury
*T*_c_: core temperature
TNF: tumour necrosis factor
